# The mediating role of diabetes stigma and self-efficacy in relieving diabetes distress among patients with type 2 diabetes mellitus: a multicenter cross-sectional study

**DOI:** 10.3389/fpsyg.2023.1147101

**Published:** 2023-07-28

**Authors:** Shuping Xing, Yeling Liu, Hua Zhang, Bin Li, Xinjun Jiang

**Affiliations:** ^1^International Nursing School, Hainan Medical University, Haikou, Hainan, China; ^2^International Medical Service Centre, The First Affiliated Hospital of Shantou University Medical College, Shantou, China; ^3^Department of Nursing Management, Hainan General Hospital, Haikou, Hainan, China

**Keywords:** type 2 diabetes mellitus, diabetes distress, diabetes stigma, social support, diabetes self-efficacy

## Abstract

**Background:**

Patients with diabetes mellitus often suffer from diabetes distress. Social support and certain psychological factors potentially influence diabetes distress, but studies exploring the mechanisms underlying these relationships are scarce.

**Objectives:**

To reveal the associations between social support, diabetes stigma, diabetes self-efficacy, and diabetes distress among patients with type 2 diabetes and the underlying mechanisms linking these variables.

**Design and methods:**

A multicenter cross-sectional study was adopted and a sample of 431 patients with type 2 diabetes was investigated. Social support, diabetes stigma, diabetes self-efficacy, and diabetes distress were surveyed with the Perceived Social Support Scale, Type 2 Diabetes Stigma Assessment Scale, Self-Efficacy for Diabetes Scale, and Diabetes Distress Scale, respectively. The hypothesized model was verified using structural equation modeling.

**Results:**

Social support and diabetes stigma had direct associations with diabetes distress. Diabetes stigma mediated the association between social support and diabetes distress, and the association between diabetes self-efficacy and diabetes distress. Diabetes stigma and self-efficacy exerted a chain mediation effect on the association between social support and diabetes distress.

**Conclusion:**

Social support and diabetes stigma were significant predictors of diabetes distress. Diabetes stigma and self-efficacy play essential mediating roles in relieving diabetes distress. This can provide guidance for the development of evidence- and theory-based interventions. Culturally sensitive interventions that aim to provide ongoing social support, decrease diabetes stigma, and enhance self-efficacy have the potential to relieve diabetes distress.

## Introduction

Diabetes mellitus poses a severe threat to human health, with over 90% of patients diagnosed with type 2 diabetes mellitus (T2DM) worldwide (International Diabetes Federation, 2021). Diabetes mellitus can cause a range of psychological problems (e.g., diabetes distress) in patients due to chronic disease progression, the development of diabetes-related complications, and the necessity of life-time disease management. Diabetes distress is defined as negative psychological responses to emotional burdens and excessive concerns about the experience of a patient in managing diabetes mellitus and preventing complications (Fisher et al., 2012; American Diabetes Association Professional Practice Committee, 2022).

Diabetes distress is very common in patients with diabetes mellitus. For instance, several cross-sectional studies have found that the prevalence of diabetes distress ranges from 23.7 to 68.5% (Zhou et al., 2017; Azadbakht et al., 2020; Niroomand et al., 2021; Presley et al., 2021). Moreover, a scoping review of 46 studies on diabetes distress in South Asian adults living in developing countries found that the incidence of diabetes mellitus varied between 18.0 and 76.2% (Kalra et al., 2020). Furthermore, a systematic review revealed that 36% of T2DM patients experienced diabetes distress (Perrin et al., 2017). A high level of diabetes distress often significantly affects diabetes-related self-management behaviors such as non-adherence to medication, dietary adjustment, and healthcare use (Zhang et al., 2021), which could result in poor glycemic control outcomes (e.g., high HbA1c levels; Niroomand et al., 2021; Schmitt et al., 2021). In addition, diabetes distress can reduce work and life productivity (Xu et al., 2020). According to diabetes guidelines distributed by American Diabetes Association (American Diabetes Association Professional Practice Committee, 2022), it is recommended that diabetes distress should be routinely monitored among patients with diabetes. Therefore, determining the factors might aggravate diabetes distress in T2DM patients is essential.

## Conceptual model

Factors influencing diabetes distress might arise from environmental and psychological aspects because it is a psychological problem. According to Social Cognitive Theory (SCT), environmental factors (e.g., social support) are closely related to individual psychological factors (e.g., diabetes distress, self-efficacy, and diabetes stigma; Bandura, 1986). But the empirical relationships are unclear among the environmental factors and the individual psychological factors, and relationships among variables of psychological factors. Social support refers to one’s perception of receiving support and assistance from various sources such as family members, friends, and other social contacts (Su et al., 2012). Social support is vital to support patients with diabetes mellitus in terms of daily emotional adjustment and disease management. According to some studies, social support is closely associated with psychological indicators such as diabetes distress (Chan et al., 2020; Beverly et al., 2021; Geleta et al., 2021; Presley et al., 2021). Therefore, social support is likely to affect diabetes distress.

Negative psychological indicators of diabetes stigma might exert an effect on diabetes distress. Diabetes stigma is defined as a negative perception related to stereotyping, criticism, rejection, and refusal due to the disease or its management (Browne et al., 2013; Winkley et al., 2015). Research on diabetes stigma remains in its early stages, and qualitative studies suggest that diabetes stigma may place psychological pressure on diabetes patients and impair their disease management efforts (Browne et al., 2013, 2014; Winkley et al., 2015). One quantitative study found that diabetes stigma hindered patients with diabetes from participating in self-management education programs (Yan et al., 2022). Therefore, diabetes stigma is suspected to be associated with diabetes distress, although empirical evidence is required to support this argument. In addition, some studies indicate that diabetes stigma might be associated with social support (Holmes-Truscott et al., 2020; Weaver et al., 2022). Therefore, diabetes stigma may exert a direct effect on diabetes distress and mediate the association between social support and diabetes distress.

As one of the most significant individual psychological factors, diabetes self-efficacy indicates one’s confidence in the ability to conduct diabetes management activities in daily life (Hurley and Shea, 1992). Research has found that social support affects diabetes self-efficacy (Chan et al., 2020; Al-Dwaikat et al., 2021; Yang et al., 2021). Furthermore, a study conducted in China suggested that diabetes self-efficacy might mediate the association between social support and diabetes stigma in T2DM patients (Wang et al., 2020). Thus, diabetes self-efficacy could exert a mediation effect between social support and diabetes stigma and influence diabetes distress through diabetes stigma. However, further research is required to confirm this hypothesis.

**Figure 1:**
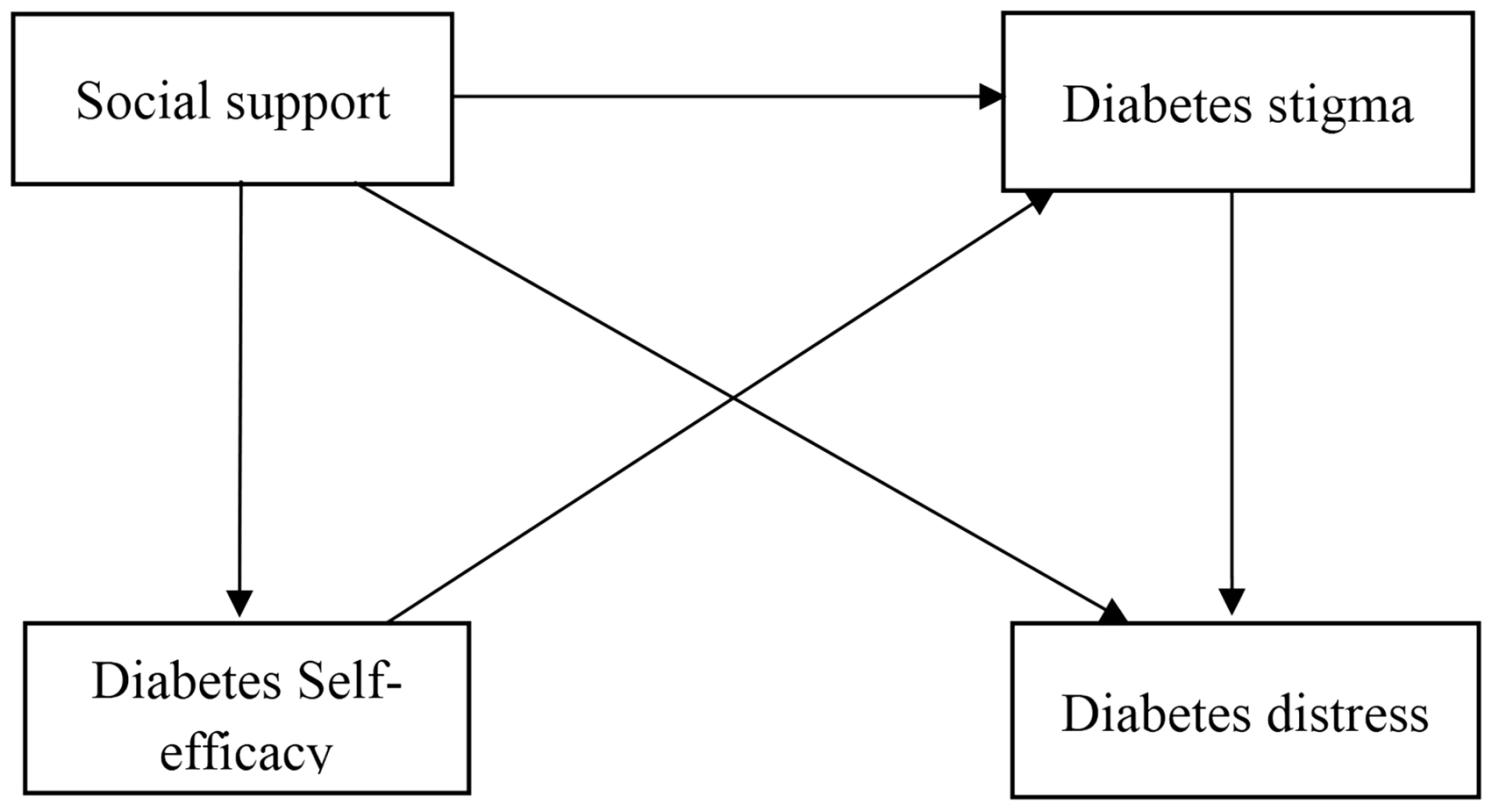
The hypothesized model of the effects of social support and self-efficacy on diabetes stigma and diabetes distress.

In summary, the prevalence of diabetes distress is relatively high among T2DM patients. Diabetes distress can hinder patients’ self-management, resulting in poorer glycemic control. There is currently insufficient evidence of how environmental factors are related to individual factors, and the aforementioned studies have suggested that diabetes distress might be affected by various factors including social support, self-efficacy, and diabetes stigma. Moreover, most studies have focused only on the bivariate relationships of variables, and there are few studies on the underlying mechanisms among these variables. Therefore, it is necessary to further explore the relationships between these variables, including social support, self-efficacy, diabetes stigma, and diabetes distress. Figure 1 shows a conceptual model developed based on SCT and the aforementioned findings of prior studies. The aim of this study is to reveal the associations between social support, diabetes stigma, self-efficacy, and diabetes distress in T2DM patients.

## Methods

### Participants

The STROBE checklist of observational studies (Supplementary Appendix S1) was adopted to guide for the report of the study. Using convenience sampling, a multicenter, cross-sectional study was conducted in the endocrinology departments of three tertiary hospitals in Hainan Province from August 2020 to March 2021. The participants in this study were adults with T2DM. There were two inclusion criteria: the patient was aged no less than 18 years, and was willing to participate in the study. Patients who had mental health problems, cognitive disorders, or had participated in other studies were excluded. The sample size of this study was determined by the number of parameters tested, and the minimum sample size was set to 200 for structural equation modeling purposes (Wu, 2023). A minimum ratio of sample size to parameters of (10–15):1 was recommended by Thompson (2000). Nine parameters were observed in the study. Therefore, the required sample size was determined to be 135, according to a ratio of 15:1. A sample size of 200 was adopted, given the minimum sample size for structural equation modeling. The final sample for recruitment was set to 236 after considering a 15% dropout rate.

### Indicators and instruments

The participants’ characteristics and the four variables of social support, diabetes self-efficacy, diabetes stigma, and diabetes distress were investigated in this study. The questionnaire and scales included the Chinese versions of Perceived Social Support Scale (C-PSSS), Self-Efficacy for Diabetes Scale (C-SED), Type 2 Diabetes Stigma Assessment Scale (C-DSAS-2), Diabetes Distress Scale (C-DDS), and a basic information questionnaire. The PSSS, SED, DSAS-2, and DDS were developed in Western countries, while Chinese versions have undergone translation, cultural adaptation, etc., through the efforts of local scholars.

### Social support

The C-PSSS was used to assess social support. The C-PSSS was translated by Jiang (2001) based on the English version of PSSS developed by Zimet et al. (1990). The C-PSSS includes three subscales: support from family members, support from friends, and other sources of support. Each subscale consists of four items, and all of them are rated from one to seven. The total score is equal to the sum of each item, ranging from 12 to 84 points. A higher score indicates better perceived social support. Cronbach’s *α* and the concurrent validity of the C-PSSS exceeded 0.800 (Jiang, 2001), indicating that the scale was satisfactory. Cronbach’s α in the present survey was 0.954.

### Diabetes stigma

The C-DSAS-2 was used to evaluated diabetes stigma. Developed by Browne et al., this instrument was introduced in China by Li et al. (2017). The C-DSAS-2 comprises three subscales: differential treatment, blame and judgment, and self-stigma. Nineteen items are rated on five-point Likert scales. The total score can range from 19 to 95 points based on the sum of each item. A higher score suggests greater perceived diabetes stigma. Cronbach’s α and the test–retest reliability suggest that the C-DSAS-2 is a satisfactory scale (Li et al., 2017). Cronbach’s α in the current study was 0.936.

### Diabetes self-efficacy

The C-SED was adopted to measure diabetes self-efficacy. The SED was developed by experts at Stanford University (Stanford Patient Education Research Center, 2017), while the C-SED was translated and revised by Wei (2013). Consisting of 9 items, the C-SED contains four aspects: diet, physical activity, glycemic control, and disease management. A five-point Likert scale was used for each item. The mean score of the scale was calculated, and mean scores can range from 1 to 5. A high mean score indicates greater self-efficacy. The validity and reliability of the C-SED have been confirmed (Wei, 2013). Cronbach’s α in this investigation was 0.970.

### Diabetes distress

The C-DDS was adopted to assess diabetes distress. The DDS was developed by Polonsky et al. (2005); the Chinese version was developed and revised by Li (2012). The C-DDS contains four subscales: negative emotion, social support and self-management, the relationship between physician and patient, and medical resources. Based on the sum of the 17 items, the total score can range from 17 to 102. A higher score indicates greater distress. The mean item score was used to evaluate the severity of diabetes distress. A mean score of ≥3, 2 to <3, and < 2 indicates a high, moderate, or low level of diabetes distress, respectively (Fisher et al., 2012). Cronbach’s α and the criterion validity indicate that the C-DDS is a satisfactory instrument (Li, 2012). In this survey, Cronbach’s *α* was 0.914.

### Participants’ characteristics

A self-designed basic information questionnaire was used to obtain the characteristics of the participants, including demographic information (e.g., gender, age, marriage, employment status, educational background, individual monthly income, and medical insurance) and clinical information (e.g., diabetes-related complications and the duration of diabetes).

### Data collection

This survey was conducted from August 2020 to March 2021 in the endocrinology departments of three tertiary hospitals in Haikou city, Hainan Province. In general, participants completed the pen-and-paper survey independently after the introduction and explanation by the investigators. A double check was then conducted by the investigator after the questionnaires were returned to minimize the amount of missing data. If the questionnaire was incomplete, the respondent was asked to fill in the missing items immediately. Otherwise, the questionnaire was marked invalid. However, if the participants were illiterate, the investigators used a format of one question-one answer to collect data. Before the survey was conducted, the investigators received training on the basic information of the study and data collection process. Finally, 439 adults with T2DM were investigated, and 431 valid questionnaires with no missing data were recovered, with a rate of 98.2%.

### Ethical issues

This study was approved by the Ethics Committee of Hainan Medical University (HYLL-2020-010). Prior to the study, patients with T2DM were informed by the investigators about the research aims, significance, procedures, and other relevant information about the study. Additionally, patients were entitled to withdraw at any time, and the data collecting from the patients were stored securely by the research group to maintain confidentiality and anonymity.

### Data analysis

The software SPSS (version 25.0, IBM Corp.) was adopted to perform data analysis. The normality of participant characteristic distributions and key variables was checked using the Kolmogorov–Smirnov test. The mean and standard deviation (SD) were used to display the normally distributed continuous variables. Otherwise, the median and the upper and lower quartiles were used. The frequency and percentage were used to present the categorical variables. The Cronbach’s *α* of each instrument was determined, and Pearson correlation analysis was carried out to test the correlations among the four key variables, namely, social support, self-efficacy, diabetes stigma, and diabetes distress. *p* < 0.05 was considered the statistical significance.

In addition, structural equation modeling was performed using AMOS (version 24.0, IBM Corp.) to test the hypothesized model using the maximum likelihood estimation method. Bias-corrected bootstrap 95% confidence intervals (CIs) were used in combination with 10,000 iterations to evaluate the significance of the indirect effect and 95% CI of path coefficients. The fitness of the model was assessed in accordance with the following criteria: (a) chi-square (χ^2^) with *p* > 0.05; (b) ratio of χ^2^ and degree of freedom (DF) below 3; (c) NFI > 0.90, GFI > 0.90, and RMSEA <0.08 (Hair et al., 1998; Byrne, 2009).

## Results

### Participants’ characteristics

As displayed by Table 1, the mean age of the 431 patients was 58.74 years (*SD* = 11.63); 39.44% of the participants were male, and 93.04% were married. Nearly two-thirds of the participants (65.20%) were retired, 27.15% had an elementary school education and below, over half (53.13%) earned an individual income of no greater than 2000 Chinese Yuan, and 64.73% were covered by the urban employee or residential insurance. On average, the duration of diabetes since diagnosis was 8.28 years (*SD* = 6.96). Moreover, 38.05% of participants reported developing diabetes-related complications.

**Table 1:** Participants’ characteristics (*N* = 431).

Variables	Classification	Number (*n*)	Proportion (%)	Mean (*SD*)
Age (years)	<60	208	48.26	58.74 (11.63)
	≥60	223	51.74	
Gender	Male	170	39.44	
	Female	261	60.56	
Marriage	None or others	30	6.96	
	Married	401	93.04	
Employment status	Employed	150	34.80	
	Retired	281	65.20	
Education background	Elementary school and below	117	27.15	
	Middle school and above	314	72.85	
Individual monthly income (CNY)	≤2000	229	53.13	
	>2000	202	46.87	
Medical insurance	Urban employee or residential insurance	279	64.73	
	Cooperative medical scheme or others	152	35.27	
Duration of diabetes (year)	≤5	194	45.01	
	>5	237	54.09	
Diabetes complication	Yes	164	38.05	
	No or unclear	267	61.95	

CNY, Chinese Yuan; SD, standard deviation.

### Levels of variables and their correlations

The proportion of participants with moderate or severe diabetes distress (moderate-to-severe) was 25.29%. Table 2 shows the descriptive statistics, intercorrelations between the variables, and reliability values of the scales. The mean scores of social support, diabetes self-efficacy, diabetes stigma, and diabetes distress were 65.74 (*SD* = 11.44), 3.39 (*SD* = 1.15), 41.74 (*SD* = 12.57), and 27.14 (*SD* = 9.12), respectively. Significant pairwise correlations were observed between the measured variables.

**Table 2:** Levels of variables and the correlations among the variables (*N* = 431).

Variables	Mean *(SD)*	Min.	Max.	1	2	3	4	Cronbach’s *α*
1. Social support	65.74 (11.44)	12	84	1				0.954
2. Self-efficacy	3.39 (1.15)	1	5	0.505***	1			0.970
3. Diabetes stigma	41.74 (12.57)	19	81	−0.485***	−0.531***	1		0.936
4. Diabetes distress	27.14 (9.12)	17	81	−0.313***	−0.270***	0.309***	1	0.914

*p < 0.05, **p < 0.01, ***p < 0.001. The numbers on the top of the table denote as follows: (1) social support, (2) self-efficacy, diabetes self-efficacy, (3) diabetes stigma, and (4) diabetes distress.

### Structural equation modeling

**Figure 2:**
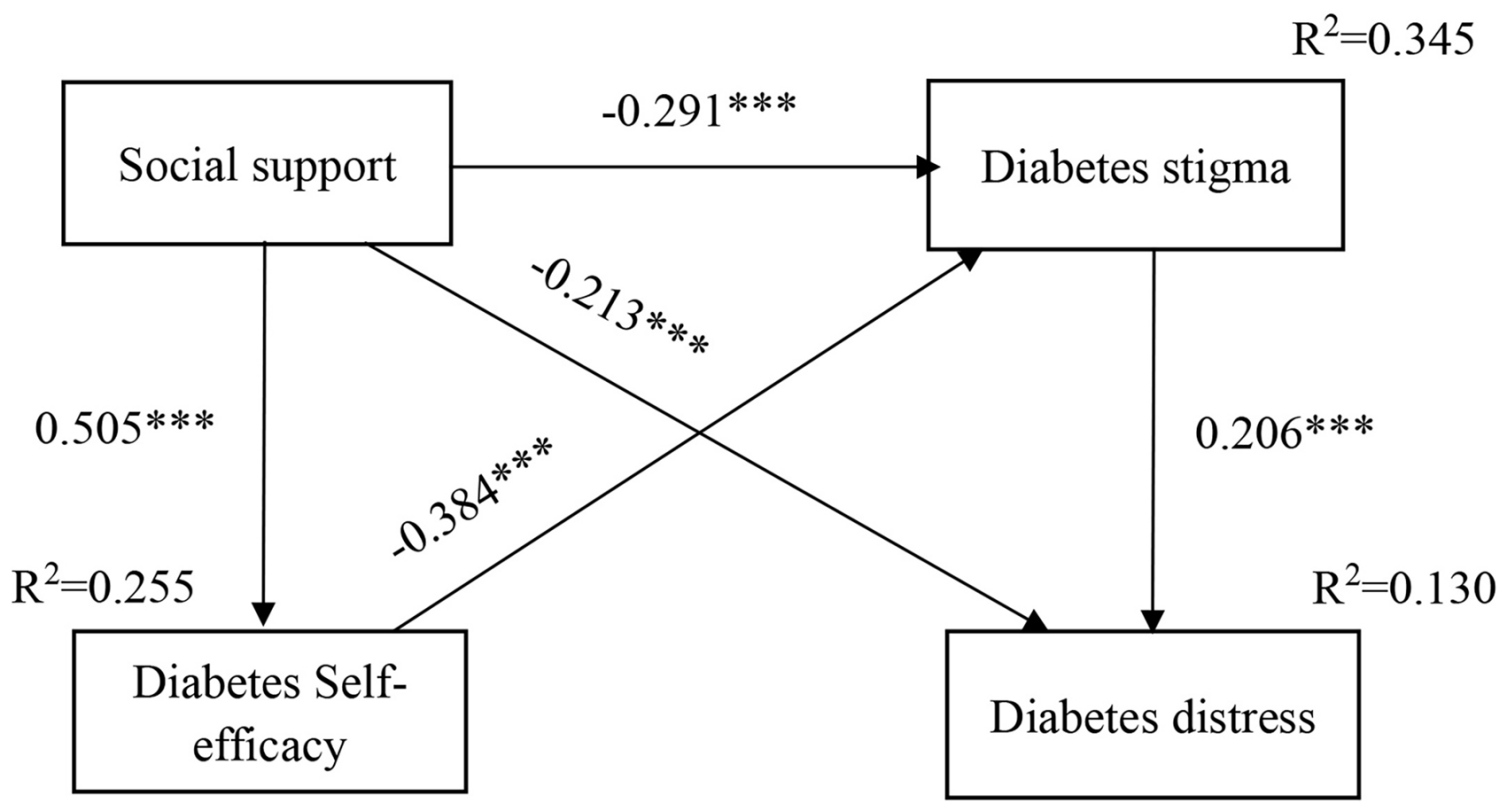
The model with standardized estimates of social support, self-efficacy, diabetes stigma and diabetes distress (***p < 0.001).

There was a good fit to the data, as shown by the structural model that hypothesized the associations between the key variables, with all paths in the structural model showing significance (Figure 2). Regarding the direct effects, social support had negative associations with diabetes distress and stigma, but had positive associations with self-efficacy. Diabetes stigma had a positive association with diabetes distress. In addition, diabetes self-efficacy had a negative relationship with diabetes stigma.

Regarding the indirect effects, diabetes stigma, by itself and in combination with self-efficacy, had mediation effects on the association between social support and diabetes distress, which explained 31.9% of the total effect of social support on diabetes distress. Diabetes stigma played a complete mediating role in the association between self-efficacy and diabetes distress. Self-efficacy exerted a partial effect on the relationship between social support and diabetes stigma, which explained 40% of the total effect exerted by social support on diabetes stigma.

In total, 13.0% of the variance for diabetes distress was accounted for by social support, diabetes self-efficacy and diabetes stigma, while 34.5% of the variance for diabetes stigma was explained by social support and diabetes self-efficacy. Moreover, 25.5% of the variance in the diabetes self-efficacy was explained by social support.

## Discussion

A multicenter cross-sectional study demonstrated the direct effects of diabetes distress exerted by diabetes stigma and social support, and revealed the separate mediating role of diabetes stigma and the chain mediating role of diabetes stigma and self-efficacy in relieving the diabetes distress. This finding can provide guidance for the development of evidence- and theory-based interventions for T2DM patients.

Patients with T2DM in this multicenter cross-sectional study had relatively low mean scores on diabetes distress, and the prevalence of diabetes distress was also low, which was clearly better than the results of several previous studies conducted both in China and internationally (Fisher et al., 2012; Zhou et al., 2017; Jiang et al., 2019a; Azadbakht et al., 2020; Niroomand et al., 2021). This can be explained from two perspectives: On one hand, the results of social support and diabetes self-efficacy in the study were above the middle level, while diabetes stigma appeared to be below the middle level. Additionally, these factors affected diabetes distress directly and indirectly. Therefore, relatively good levels of social support, diabetes management confidence, and low levels of diabetes stigma in T2DM patients can contribute to alleviating diabetes distress. On the other hand, the population investigated in this study is from Hainan Island, which is the second biggest island in China. Located in an area with an oceanic tropical monsoon climate, Hainan Island is an international tourist island. The service industry, as represented by tourism, is a central pillar of the local economy. Currently, this island lags behind most other Chinese provinces in terms of economic development. Thus, people, including patients on this island, have a relatively more leisurely and comfortable lifestyle rather than having a rapid work pace and life rhythm. Such circumstances are conducive to relieving various negative emotions, such as diabetes distress in T2DM patients. Despite this, it is necessary to routinely monitor diabetes distress in patients in clinics.

According to the results of this study, high levels of social support were correlated with low levels of diabetes distress, which is basically consistent with the findings of previous studies (Chan et al., 2020; Beverly et al., 2021; Geleta et al., 2021; Presley et al., 2021). The results of this study also showed that high social support predicted low diabetes stigma and high diabetes self-efficacy, which is similar to the results of other studies (Holmes-Truscott et al., 2020; Al-Dwaikat et al., 2021; Presley et al., 2021; Yang et al., 2021). The greater the social support perceived by T2DM patients suggests they receive more support from their family members, relatives, and other sources. T2DM patients receiving such support would have access to advice and other support resources, which might contribute directly to better understanding the disease, enhancing confidence about overcoming barriers to managing the disease, and alleviating negative emotions. A study revealed that better quality and function of social support were significantly related to improvement of self-efficacy and self-management in T2DM patients (Al-Dwaikat et al., 2021). Thus, patients’ perceived social support can be regarded as a pertinent factor for the alleviation of diabetes distress, and it is necessary to conduct assessments before patients are provided with social support, which might provide a more targeted solution to assisting T2DM patients. At present, diabetes self-management education is recognized as a good way to support patients with diabetes mellitus (American Diabetes Association, 2022). However, a previous systematic review suggested that the effect of diabetes self-management education tends to diminish within 12–24 months (Captieux et al., 2018). This indicates that different kinds of ongoing social support are required to help patients maintain low levels of diabetes distress.

Additionally, this study found some other interesting results. Diabetes stigma mediated the relationship between social support and diabetes distress. It also mediated the relationship between self-efficacy and diabetes distress. Another interesting finding was that both diabetes self-efficacy and diabetes stigma played a chain-mediating role in the association between social support and diabetes distress. The results showed that the indirect effects of diabetes stigma and self-efficacy accounted for 31.9% of the total effect of social support on diabetes distress. Compared with the previous studies mentioned earlier, the results obtained in this study provide firm evidence of the mediating roles played by self-efficacy and diabetes stigma in the association between social support and diabetes distress. Thus, to help patients further relieve their diabetes distress, decreasing diabetes stigma, and enhancing self-efficacy can be considered as targets for intervention.

The results of the study suggested that diabetes stigma not only played a mediating role in relieving diabetes distress but also exerted a direct effect on diabetes distress. Thus, the assessment and alleviation of diabetes stigma must be considered when implementing interventions. However, research on diabetes stigma is relatively new and recent research on interventions for diabetes stigma is scarce. Diabetes stigma includes self-stigma, such as feelings of guilt and failure, and perceptions of blame, judgment, and differential treatment from family members, colleagues, and even medical staff due to the disease (Browne et al., 2016). Thus, interventions aimed at improving diabetes stigma related to cognition and response abilities and providing support from family members and medical staff might have the potential to relieve diabetes stigma and diabetes distress in patients with T2DM. In addition, a World Health Organization language survey found that the media often convey messages that diabetes mellitus was caused by poor lifestyle, habits, and deficiencies, and these can lead to patients being blamed by the public, which further increases patients’ stigma and stereotypes (Hunt et al., 2022). Thus, the media should use standard, non-judgmental, evidence-based, and inclusive reporting language to create more effective publicity and education about diabetes mellitus for the public. This would help to create a more supportive environment, improve stereotypes about the disease, and reduce discrimination against patients with diabetes mellitus. This will ultimately contribute to alleviating diabetes stigma, decreasing distress, and prompting active self-management.

Regarding intervention for self-efficacy, a systematic review suggested that self-efficacy-focused education can improve glycemic control, self-efficacy, and self-management behaviors (Jiang et al., 2019b). In a real word clinical trial conducted by Jiang et al. (2019a, 2021) to deliver a self-efficacy-focused structured education program in mainland China, it was suggested that improvements in diabetes self-efficacy, diabetes distress, glycemic control, and other metabolic and psychosocial aspects could be achieved in 6–12 months for T2DM patients. Therefore, it is possible to improve self-efficacy and alleviate diabetes distress through a diabetes self-management education program focusing on the enhancement of diabetes self-efficacy that organically combines goal setting, positive feedback, experience sharing, peer support, etc. (Jiang et al., 2019a, 2021). Additionally, one study found that diabetes distress could be reduced by collaborative goal-setting with enhanced education (Woodard et al., 2022). Thus, interventions focusing on increasing diabetes self-efficacy, integrated with strategies such as reasonable goal setting, positive feedback, experience sharing, and peer support, are likely to be applicable in reducing diabetes distress.

In addition, the study indicated that self-efficacy was negatively associated with diabetes stigma and that it mediated the association between social support and diabetes stigma, which is consistent with the results obtained by Wang et al. (2020). Patients with high levels of diabetes self-efficacy can be more confident in resisting the pressure and burden of the disease (such as self-management aspects, perceptions of blame and judgment from family members, concerns about being treated differently, etc.), and in overcoming the difficulties encountered in daily life. Consequently, diabetes stigma can be reduced. Moreover, a high level of social support indicated that patients could gain understanding and support from family members, friends, and colleagues, which would boost their confidence, thereby further reducing diabetes stigma.

In this study, the hypothesized model explained 13.0, 25.5, and 34.5% of the variation in diabetes distress, diabetes stigma, and self-efficacy, respectively. The explanatory strength of the variables in the model is acceptable, according to Cohen (1988). This multicenter cross-sectional study provides strong evidence to suggest that social support and diabetes stigma are directly associated with diabetes distress and that self-efficacy and diabetes stigma play important mediating roles in influencing diabetes distress. These findings provide insights into the relationships among the variables, including social support, diabetes self-efficacy, diabetes stigma, and diabetes distress among patients with T2DM. Additionally, the study provides empirical evidence for the principle of SCT that the mechanism includes both the environmental factors of social support and individual factors of diabetes self-efficacy, diabetes stigma, and diabetes distress. Furthermore, since this study was conducted in Hainan province, which is a tropical area, the findings of this study might be extrapolated to other tropical regions/areas.

Nevertheless, this study has some limitations. First, because convenience sampling was performed, the results obtained in this study must be interpreted cautiously. Second, the participants in the study were recruited from three hospitals that were all located in Hainan province on account of finite resources, which means it should be caution to generalize the results widely. More research is needed to verify whether the findings are applicable to different provinces and regions. Nevertheless, it still can provide information for participants from different provinces and regions. Third, it is potentially difficult to infer causal relationships between social support, self-efficacy, diabetes stigma, and diabetes distress in this cross-sectional study. A longitudinal study can be designed to further explore these relationships.

## Conclusion

The results of the model illuminate theoretical mechanisms underlying the variables of social support, diabetes stigma, self-efficacy, and diabetes distress in T2DM patients. As demonstrated by the model, diabetes stigma mediated the associations between social support and diabetes distress as well as between self-efficacy and diabetes distress, while self-efficacy mediated the association between social support and diabetes stigma. Diabetes stigma and self-efficacy played a chain-mediating role in the association between social support and diabetes distress. Diabetes distress was directly affected by social support and diabetes stigma. The results of the model can provide guidance for the development of evidence- and theory-based interventions. By providing social support, enhancing diabetes self-efficacy, and relieving diabetes stigma, self-management interventions might help alleviate diabetes distress in T2DM patients.

## Data availability statement

The data analyzed in this study is subject to the following licenses/restrictions: the original contributions presented in the study are included in the article, further inquiries can be directed to the corresponding authors. Requests to access these datasets should be directed to XJ, Jxinjun@hainmc.edu.cn.

## Ethics statement

The studies involving human participants were reviewed and approved by the Ethics Committee of Hainan Medical University (HYLL-2020-010). The patients/participants provided their written informed consent to participate in this study.

## Author contributions

SX and YL contributed to the design of the study, data collection and analysis. XJ, BL, and HZ conceptualized and supervised the study. XJ, SX, YL, BL, and HZ prepared and revised the manuscript. All authors prepared the manuscript and approved the final version for submission.

## Funding

This study was supported by the Hainan Provincial Natural Science Foundation of China (820RC631), Young Talents’ Science and Technology Innovation Project of Hainan Association for Science and Technology (QCXM202019), Key Research and Development Project in Hainan Province (ZDYF2022SHFZ102), the Project of Science Research Project in Hainan University of Higher Education (Hnky2020-36), and Hainan Health Commission Health Industry Research Project (21A200237).

## Conflict of interest

The authors declare that the research was conducted in the absence of any commercial or financial relationships that could be construed as a potential conflict of interest.

## Publisher’s note

All claims expressed in this article are solely those of the authors and do not necessarily represent those of their affiliated organizations, or those of the publisher, the editors and the reviewers. Any product that may be evaluated in this article, or claim that may be made by its manufacturer, is not guaranteed or endorsed by the publisher.
