# Supplementary material for: The mediating role of diabetes stigma and self-efficacy in relieving diabetes distress among patients with type 2 diabetes mellitus: a multicenter cross-sectional study
Source: Front Psychol. 2023 Jul 28;14:1147101. doi: 10.3389/fpsyg.2023.1147101 (PMC10416640; doi:10.3389/fpsyg.2023.1147101)
Supplement: Supplementary file 1 [file Data_Sheet_1.PDF]

STROBE Statement—Checklist of items that should be included in reports of ***cross-sectional studies***

	Item No	Recommendation
**Title and abstract**	1	(*a*) Indicate the study's design with a commonly used term in the title or the abstract (Yes, page 1-2)
		(*b*) Provide in the abstract an informative and balanced summary of what was done and what was found (Yes, abstract section, Page 1-2)
**Introduction**		
Background/rationale	2	Explain the scientific background and rationale for the investigation being reported (Yes, introduction section, Page 3-5)
Objectives	3	State specific objectives, including any prespecified hypotheses (Yes, introduction section, Page 5)
**Methods**		
Study design	4	Present key elements of study design early in the paper (Yes, study design and participants section, Page 5-6)
Setting	5	Describe the setting, locations, and relevant dates, including periods of recruitment, exposure, follow-up, and data collection (Yes, study design and participants section, Page 5-6)
Participants	6	(*a*) Give the eligibility criteria, and the sources and methods of selection of participants (Yes, study design and participants section, Page 5-6)
Variables	7	Clearly define all outcomes, exposures, predictors, potential confounders, and effect modifiers. Give diagnostic criteria, if applicable (Yes, indicators and instruments section, Page 6-8)
Data sources/ measurement	8*	For each variable of interest, give sources of data and details of methods of assessment (measurement). Describe comparability of assessment methods if there is more than one group (Yes, indicators and instruments section, Page 6-8)
Bias	9	Describe any efforts to address potential sources of bias (Yes, data collection section, Page 8-9)
Study size	10	Explain how the study size was arrived at (Yes, study design and participants section, Page 6, and data collection section, Page 8-9)
Quantitative variables	11	Explain how quantitative variables were handled in the analyses. If applicable, describe which groupings were chosen and why (Yes, data analysis section, Page 9)
Statistical methods	12	(*a*) Describe all statistical methods, including those used to control for confounding (Yes, data analysis section, Page 9)
		(*b*) Describe any methods used to examine subgroups and interactions Not applicable.
		(*c*) Explain how missing data were addressed Not applicable.
		(*d*) If applicable, describe analytical methods taking account of sampling strategy Not applicable.
		(*e*) Describe any sensitivity analyses Not applicable.

**Results**		
Participants	13*	(a) Report numbers of individuals at each stage of study—eg numbers potentially eligible, examined for eligibility, confirmed eligible, included in the study, completing follow-up, and analysed<br>Not applicable.
		(b) Give reasons for non-participation at each stage<br>Not applicable.
		(c) Consider use of a flow diagram<br>None, we used texts to illustrate this.
Descriptive data	14*	(a) Give characteristics of study participants (eg demographic, clinical, social) and information on exposures and potential confounders<br>(Yes, results section, Page 10, and table 1)
		(b) Indicate number of participants with missing data for each variable of interest<br>Not applicable.
Outcome data	15*	Report numbers of outcome events or summary measures<br>(Yes, results section, Page 10-11, and table 1-2)
Main results	16	(*a*) Give unadjusted estimates and, if applicable, confounder-adjusted estimates and their precision (eg, 95% confidence interval). Make clear which confounders were adjusted for and why they were included<br>(Yes, results section, Page 10-11, table 1-2, and figure 1-2)
		(*b*) Report category boundaries when continuous variables were categorized<br>(Yes, results section, Page 10-11, table 1-2)
		(*c*) If relevant, consider translating estimates of relative risk into absolute risk for a meaningful time period<br>Not applicable.
Other analyses	17	Report other analyses done—eg analyses of subgroups and interactions, and sensitivity analyses<br>Not applicable.
**Discussion**		
Key results	18	Summarise key results with reference to study objectives<br>(Yes, discussion section, Page 11)
Limitations	19	Discuss limitations of the study, taking into account sources of potential bias or imprecision. Discuss both direction and magnitude of any potential bias<br>(Yes, discussion section, Page 16)
Interpretation	20	Give a cautious overall interpretation of results considering objectives, limitations, multiplicity of analyses, results from similar studies, and other relevant evidence<br>(Yes, discussion section, Page 11-16)
Generalisability	21	Discuss the generalisability (external validity) of the study results<br>(Yes, discussion section, Page 11-16)
**Other information**		
Funding	22	Give the source of funding and the role of the funders for the present study and, if applicable, for the original study on which the present article is based<br>(Yes, title page, Page 18)

*Give information separately for exposed and unexposed groups.

Note: An Explanation and Elaboration article discusses each checklist item and gives methodological background and published examples of transparent reporting. The STROBE checklist is best used in conjunction with this article (freely available on the Web sites of PLoS Medicine at http://www.plosmedicine.org/, Annals of Internal Medicine at http://www.annals.org/, and Epidemiology at http://www.epidem.com/). Information on the STROBE Initiative is available at www.strobe-statement.org.
